# Supplementary material for: Edge-driven nanomembrane-based vertical organic transistors showing a multi-sensing capability
Source: Nat Commun. 2020 Feb 12;11:841. doi: 10.1038/s41467-020-14661-x (PMC7016126; doi:10.1038/s41467-020-14661-x)
Supplement: Supplementary file 1 — Supplementary Information [file 41467_2020_14661_MOESM1_ESM.pdf]

Edge-driven nanomembrane-based vertical organic transistors showing a multi-sensing capability

Ali Nawaz[1], *Leandro Merces*[1], *Denise M. de Andrade*[1,2], *Davi H. S. de Camargo*[1,3,] *and Carlos C. Bof Bufon*[1,3]*

[1] Brazilian Nanotechnology National Laboratory (LNNano), Brazilian Center for Research in Energy and Materials (CNPEM), 13083-970 Campinas, São Paulo, Brazil

[2] Department of Materials Engineering, Ponta Grossa State University (UEPG), 84030-900 Ponta Grossa, Paraná, Brazil

[3] Postgraduate Program in Materials Science and Technology (POSMAT), São Paulo State University (UNESP), 17033-360 Bauru, São Paulo, Brazil

*corresponding author: cesar.bof@lnnano.cnpem.br

Supplementary Figure

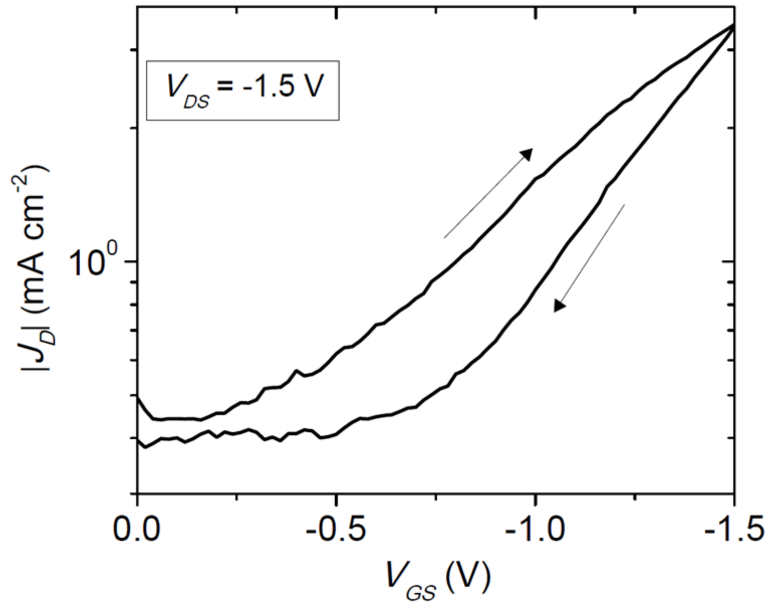

Supplementary Figure 1 Rolled-up NM-based VOFETs with circularly perforated source-electrode. Transfer curve (J_D *vs.* V_{GS}) in a logarithmic scale of devices whose electrical characteristics are shown in Figure 4 of the main text. In these devices the source-electrode was patterned with circular perforations, while the transistor dimensions and effective contact

area of these devices are as follows: t_{CuPc} = 50 nm, a = 5.43 × 10^{-5} cm, W = 0.012 cm, A_{geo} ≈ 6.5 × 10^{-7} cm^2, and A_{elect} ≈ 6.5 × 10^{-9} cm^2.

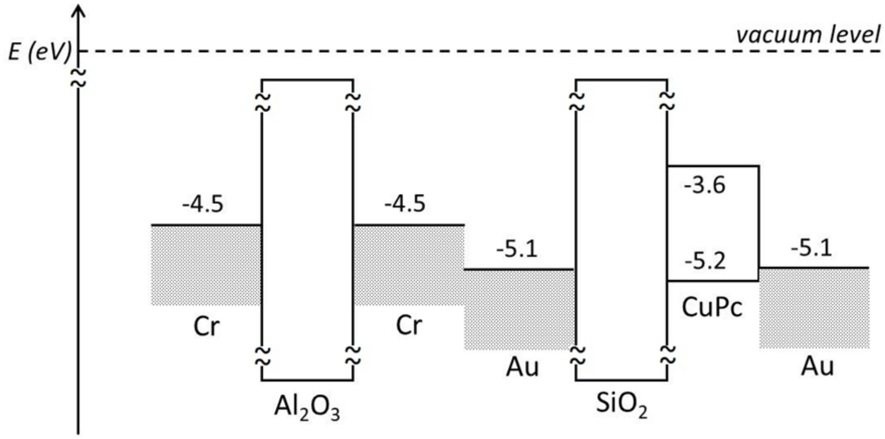

Supplementary Figure 2 Energy diagram of the rolled-up NM-based VOFET.

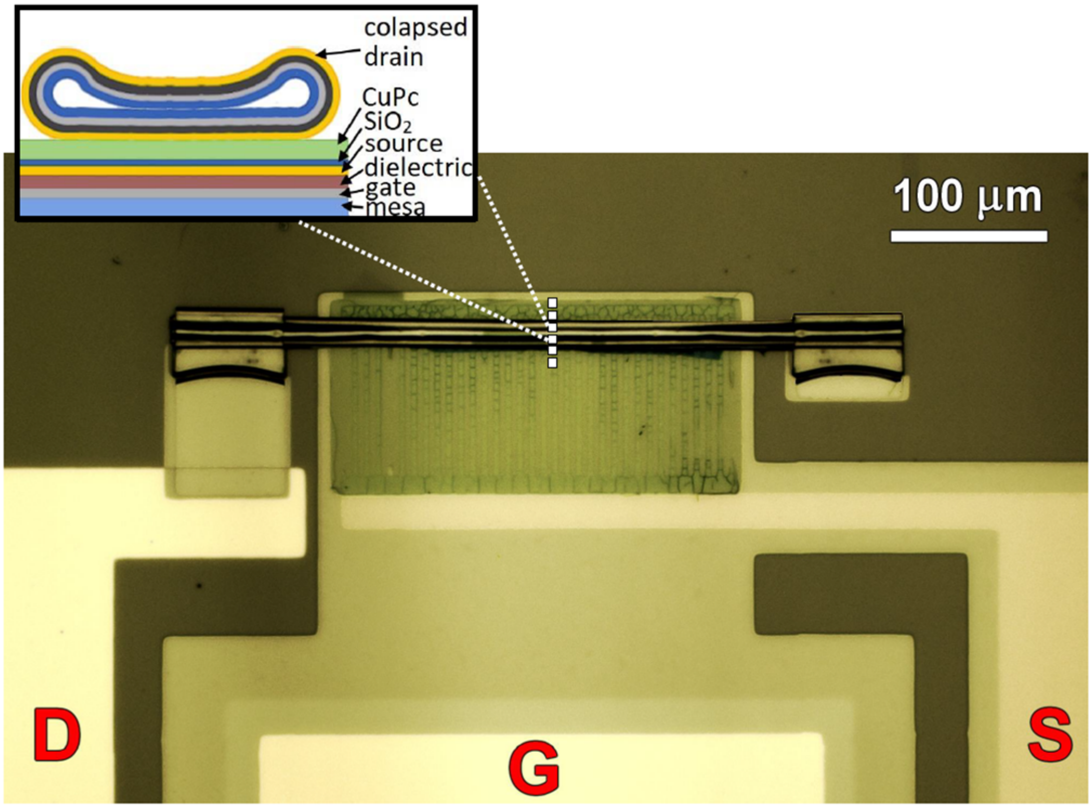

Supplementary Figure 3 Optical microscopy image of a collapsed rolled-up drain electrode. The collapsion is induced by fast sample drying after the roll-up process, which results in an irreversible compression of the tube thus modifying its shape from cylindrical to folded sheet.

A cross-sectional view of the collapsed drain (as a folded sheet) on top of the CuPc layer is illustrated in the inset.
